# Editorial: Surgical Infections

**DOI:** 10.3389/fsurg.2018.00013

**Published:** 2018-02-21

**Authors:** Evangelos P. Misiakos, Konstantinos George Tsalis

**Affiliations:** ^1^3^rd^ Department of Surgery, Medical School, National and Kapodistrian University of Athens, Attikon University Hospital, Athens, Greece; ^2^4^th^ Surgical Department, Medical School, Aristotle University of Thessaloniki, General Hospital "G. Papanikolaou", Thessaloniki, Greece

**Keywords:** surgical infections, bacteria, culture, necrosis, surgery

Surgical infections represent a common and serious problem in everyday clinical practice. Surgical site infections, after elective or emergency gastrointestinal or vascular operations, is a commonly encountered problem associated with considerable morbidity and mortality in the surgical patient (1, 2). In addition, idiopathic necrotic infections, such as necrotizing fasciitis, carry high mortality rates if surgery is delayed (3). Therefore, we decided to create a Research Topic dealing with this peculiar clinical problem. Since this is just the beginning, the published articles, so far, cannot cover all aspects of this Topic, i.e., biology, mechanisms, prevention and treatment of surgery-related infections. This Research Topic contains articles, originating from Greece, which cover several aspects of surgical infections.

 The first article is a commentary on the article (4). In this commentery, titled “Commentary: Evidence for Replacement of an Infected Synthetic by a Biological Mesh in Abdominal Wall Hernia Repair” and written by Tampaki et al., 2017, the authors state that the use of biological mesh in difficult hernias with infected mesh seems to be of clinical benefit. However, the increased expenses associated with its use cannot be fully justified, as there is not sufficient evidence from randomized trials proving their advantages.

 The second article is also a commentary but on (5). This commentary titled, “Commentary: Hernia, Mesh, and Topical Antibiotics, Especially Gentamycin: Seeking the Evidence for the Perfect Outcome…” is also written by Tampaki et al., 2017, from the Univerity of Athens. The aim of this paper is to study the impact of prophylactic use of topical antibiotics, especially gentamycin, in hernia repair with mesh. The authors agree with Dr. Kulacoglou that gentamycin empirically used as monotherapy in hernia repair with mesh may be beneficial in patients with compromised immune status, such as advanced age, chemotherapy, comorbidities, long duration of operation, etc. They conclude that prophylactic use of antibiotics in the nosocomial environment may help reducing surgical site infections in clean surgeries, such as hernia repair with mesh, but one should take into account the existing increased antibiotic resistance rates.

 In the third paper, “Fournier’s Gangrene: Lessons Learned from Multimodal and Multidisciplinary Management of Perineal Necrotizing Fasciitis” by Ioannidis et al., 2017, the authors present their experience in the management of a potentially lethal disease, Fournier’s gangrene, in a University Surgical Depertment, in Thessaloniki, Greece. They analyze the clinical, laboratory data, comorbidities, bacterial cultures, management, complications, and clinical outcome of 20 patients who had been treated surgically in their center for perineal necrotizing fasciitis. They also underline the importance of aggressive surgical debridement in the management of this challenging condition.

 In the fourth paper, “Aortic Graft Infection: Graphene Shows the Way to an Infection-Resistant Vascular Graft” by Patelis et al., 2017, from the University of Athens, the authors analyze the unique characteristics of graphene, a two-dimensional material used in aortic reconstruction. Due to its bacteriostatic and bactericidal effects, graphene may resolve the problem of aortic graft infections in the future. Indeed, graphene and its derivatives could potentially lead the way to the production of infection-resistant or bactericidal graft materials, which can be used in aortic reconstruction or other types of arterial repair, in order to avoid the development of graft infection.

 The fifth paper titled “Simultaneous Hepatic and Mesenteric Hydatid Disease—A Case Report” by Paramythiotis et al., 2017, from Thessaloniki, Greece, describes a case of a 39 year old man with intermittent abdominal pain. Abdominal CT revealed three calcified lesions, one in the liver, one adjacent to an ileal loop, and one close to the urinary bladder. Hydatid cysts in two organs may coexist in 5–13% of cases; however, a hydatid cyst in the mesentery is extremely rare. The hepatic hydatid cyst was treated with evacuation and drainage, whereas the other two ones were excised. In conclusion, hydatid cysts should be excised when feasible, or at least they should be evacuated and drained.

 The sixth paper is entitled “Incidence and Risk Factors for Organ/Space Infection after Radiofrequency-Assisted Hepatectomy or Ablation of Liver Tumors in a Single Center: More than Meets the Eye” by Karavokyros et al., 2017, from Laikon General Hospital, Athens, Greece. In this paper the authors investigate the possible risk factors for the development of organ/space infection after liver tumor resection or radiofrequency ablation, with emphasis in secondary blood stream infections. The incidence of surgical/site infections after these procedures was calculated as 16%. Admission to the ICU was found to be a statistically significant factor predisposing to the development of postoperative infections. The authors concluded that patients with compromised physical condition, or comorbidities are at greatest risk for developing postoperative infections.

 The last paper entitled “Early Diagnosis and Surgical Treatment for Necrotizing Fasciitis: A Multicenter Study” by Misiakos et al., 2017, presents a retrospective study including 62 patients treated surgically in four University Surgical Departments in Athens, Greece. This devastating infection involved the perineum in 46.8% of cases, the lower limbs in 35.55% of cases, the upper limbs and axillary region in 8.1% of cases. Septic shock occurred in 12.9% of cases and correlated strongly with mortality. The overall mortality rate was 17.7% in this series. The authors concluded that repeat surgical debridement and meticulous perioperative management is the mainstay of treatment in this potentially lethal condition.

 We believe that this is a promising beginning with the above excellent papers in this topic. We encourage the admission of new papers from other countries in this field.

## Author Contributions

Both authors discussed the articles presented in the Research Topic "Surgical Infections" and prepared the manuscript.

## Conflict of Interest Statement

The authors declare that the research was conducted in the absence of any commercial or financial relationships that could be construed as a potential conflict of interest.
